# The Role of CXC Chemokine Receptors 1–4 on Immune Cells in the Tumor Microenvironment

**DOI:** 10.3389/fimmu.2018.02159

**Published:** 2018-09-25

**Authors:** Katharina Helene Susek, Maria Karvouni, Evren Alici, Andreas Lundqvist

**Affiliations:** ^1^Department of Medicine, Karolinska Institutet (KI), Solna, Sweden; ^2^Cell Therapy Institute, Nova Southeastern University, Fort Lauderdale, FL, United States; ^3^Department of Oncology-Pathology, Karolinska Institutet (KI), Solna, Sweden

**Keywords:** chemokines, cancer immunotherapy, metastasis, NK cells, T cells, myeloid cells

## Abstract

Chemokines govern leukocyte migration by attracting cells that express their cognate ligands. Many cancer types show altered chemokine secretion profiles, favoring the recruitment of pro-tumorigenic immune cells and preventing the accumulation of anti-tumorigenic effector cells. This can ultimately result in cancer immune evasion. The manipulation of chemokine and chemokine-receptor signaling can reshape the immunological phenotypes within the tumor microenvironment in order to increase the therapeutic efficacy of cancer immunotherapy. Here we discuss the three chemokine-chemokine receptor axes, CXCR1/2–CXCL1-3/5-8, CXCR3–CXCL9/10/11, and CXCR4-CXCL12 and their role on pro-tumorigenic immune cells and anti-tumorigenic effector cells in solid tumors. In particular, we summarize current strategies to target these axes and discuss their potential use in treatment approaches.

## Introduction

Immune evasion is a hallmark of carcinogenesis (1). Tumor cells interact closely with stromal cells, immune cells and the extracellular matrix (ECM). Via complex mechanisms these communications support tumor growth, metastatic spread, and immune escape (2). A family of small chemotactic proteins, called chemokines, has key roles in these interactions. Depending on their protein sequence, and more specifically, the location of the cysteine (C) residues at their N-terminus, chemokines are subdivided into four main classes: the C-, the CC-, the CXC-, and the CX3C-chemokines (3). Irrespective of their class, chemokines signal through binding to cognate seven-transmembrane spanning G protein-coupled receptors (GPCRs), found on the migratory cells. To date, 48 chemokines and 18 signal-transducing receptors have been identified in humans. Each chemokine can activate several different receptors. Immune cell subsets differentially express chemokine receptors, which results in their selective recruitment, according to the special needs of each environment. Within the tumor microenvironment (TME), chemokine ligand secretion is often altered compared to healthy tissue. This facilitates recruitment of pro-tumorigenic immune cells such as myeloid-derived suppressor cells (MDSCs), tumor-associated neutrophils (TAN), tumor-associated macrophages (TAM), and regulatory T cells (Treg). These cells expand during tumor progression, suppress effector lymphocytes, and are associated with worse prognosis in patients with various solid malignancies (4–7). Several studies demonstrate that tumor cells secrete chemokines in an autocrine and paracrine fashion to directly promote cancer cell growth, survival and metastasis (8). Here we focus on the impact of the CXCR1/2, CXCR3, and CXCR4 chemokine axes on recruitment of pro-tumorigenic and anti-tumorigenic immune cells in solid malignancies. We highlight the role of the CXCR1/2 axis on promoting immunosuppressive cells and the impact of CXCR3 and CXCR4 axes on increasing effector cell recruitment. Furthermore, we summarize preclinical and clinical studies that shape the therapeutic potential of chemokine-targeting and their implication in combinatorial immunotherapeutic treatment approaches.

## The role of CXCR1 and CXCR2 in solid malignancies

CXCR1 and CXCR2 are expressed by several cell types, especially neutrophils, fibroblasts and vascular endothelial cells. CXCR1 and CXCR2 bind the ligands CXCL6 and CXCL8 (IL-8) with similar affinity, while binding of CXCL1, CXCL2, CXCL3, CXCL5, and CXCL7 is mediated by CXCR2 (9). Mice do not have a CXCL8 (IL-8) gene. Moreover, the gene product of murine CXCL5, called LIX, is homologous to human CXCL6 and binds both CXCR1 and CXCR2 (10). High levels of these chemokine receptors and ligands in tumor tissues and serum are correlated with worse prognosis in several tumor types, including ovarian cancer, lung adenocarcinoma, colorectal carcinoma and pancreatic ductal adenocarcinoma (PDA) (11–15). One explanation for the poor prognosis could be the preferential recruitment of pro-tumorigenic immune cells via the CXCR1/ 2 axis (summarized in Table 1). Altered signaling pathways in tumor cells can increase chemokine secretion. For instance, overexpression of the transcription factor Snail in ovarian cancer cells upregulated CXCL1, CXCL2, and CXCL5 through the NF-kB pathway and promoted MDSC recruitment (11). Snail depletion or antibody-mediated CXCR2 targeting diminished MDSC cell numbers within tumors and increased T cell and NK cell numbers (11). Similarly, CXCL1 and CXCL2 secretion by breast cancer cells resulted in increased infiltration of pro-tumorigenic myeloid cells and was further augmented by chemotherapeutic treatment, leading to chemoresistence (16). The role of CXCL5 in recruiting CXCR2^+^ MDSC and TAN has also been shown in models of renal cell carcinoma (RCC) (17), PDA (18), melanoma (19, 20), and hepatocellular carcinoma (HCC) (21). In patients with RCC, intratumoral CXCL5 and CXCL8 levels correlated with increased MDSC infiltration (17). Targeting CXCR2 reduced MDSC numbers and increased effector T cells (17). While targeting CXCR2 alone only modestly decreased tumor burden in a murine RCC model, combination with immune checkpoint inhibition significantly reduced tumor weight (17). Similarly, high CXCL5 expression was found in PDA and mediated recruitment of CXCR2^+^ neutrophils (18). Abrogation of CXCR2 diminished neutrophil infiltration and increased the ratio of effector T cells (18). In genetically modified mice that expressed human CXCL8, MDSC were efficiently recruited to the tumor site and suppressed T cell activity (22). Collectively, these data indicate that CXCR1/2 blockade reduces pro-tumorigenic immune cell infiltration and increases T and NK cell recruitment. This supports attempts to combine CXCR1/2 blockade with other immunotherapies, such as checkpoint inhibition or adoptive cell therapy. CXCR1/2 blockade also helps to overcome chemoresistance mediated by pro-tumorigenic immune cells (16, 23). It was recently shown that chemokine signaling within the TME displays high plasticity: CXCR2^+^ TAN numbers within tumor biopsies increased in PDA patients that were previously treated with an inhibitor of CCR2 (23). Inversely, depletion of TANs resulted in increased TAM numbers and only dual inhibition of both the CXCR1, CXCR2, and CCR2 axis disrupted myeloid infiltration and improved responses to chemotherapeutic treatment (23).

**Table 1:** The effect of chemokine ligands and their receptors on immune cells within the tumor microenvironment.

**Chemokine receptor**	**Chemokine (systematic name/common name)**	**Cell type**	**Role within the tumor microenvironment**	**References**
CXCR1/CXCR2	CXCL1 (GROα) CXCL2 (GROß) CXCL5 (ENA-78)	MDSC	- Targeting CXCR2 in Snail^+^ ovarian cancer xenograft models inhibits MDSC recruitment and prolongs overall survival of tumor-bearing mice	(11)
	CXCL1 (GROα) CXCL2 (GROß)	CD11b(+)Gr1(+) myeloid cells	- CXCL1 and CXCL2 are expressed by breast cancer cells and attract myeloid cells, that secrete chemokines to promote cancer cell survival	(16)
	CXCL5 (ENA-78) CXCL8 (IL-8)	MDSC	- CXCR2^+^ MDSC are recruited via CXCL5 and CXCL8 to RCC - targeting CXCR2 reduces MDSC numbers and increases T cell infiltration - Combination of CXCR2 blockade and immune-checkpoint inhibition leads to more pronounced tumor growth reduction in murine models	(17)
	CXCL5 (ENA-78)	TAN	- CXCR2^+^ TAN are recruited into PDAC along CXCL5 - CXCR2 blockade reduces TAN numbers and increases T cell numbers	(18)
	CXCL5 (ENA-78)	MDSC	- MDSC are attracted via CXCL5 in murine metastatic uveal melanoma models and enhance epithelial-mesenchymal transition (EMT) in tumor cells	(19)
	CXCL5 (ENA-78)	TAN	- Neutrophils were efficiently recruited by CXCL5 release from human melanoma cells in xenograft mouse models	(20)
	CXCL5 (ENA-78)	TAN	- CXCL5 can be induced by TGFb and Axl and promotes neutrophil recruitment toward HCC cells	(21)
	CXCL8 (IL-8)	MDSC	- MDSC are efficiently recruited to the tumor site via CXCL8 expression in genetically modified mice	(22)
	CXCL1 (GROα) CXCL3 (GROγ) CXCL5 (ENA-78) CXCL8 (IL-8)	TAN	- TANs are recruited to orthotopic pancreatic tumor sites via the CXCR2 axis; numbers of CXCR2^+^ neutrophils in pancreatic cancer patients correlate with prognosis - In an orthotopic PDAC model CXCR2 blockade prevents TAN mobilization from peripheral blood and increases effector T cell numbers in the tumor	(23)
	CXCL8 (IL-8)	NK	- Accumulation of highly cytotoxic NK cells in metastatic lymph nodes of melanoma patients	(24)
CXCR3	CXCL10 (IP-10)	Treg	- Treg recruitment via the CXCR3/CXCL10 axis increases HCC recurrence rate after liver transplantation	(25)
	CXCL11 (I-TAC)	Treg	- CXCL11 is highly expressed in colorectal cancer; similarly CXCR3^+^ regulatory T cells are abundant in CRC specimen and can be efficiently recruited *in vitro* by CXCL11	(26)
	CXCL9 (MIG)CXCL10 (IP-10)	TIL/NK	- CXCL9 and CXCL10 expression is associated with improved patient survival in advanced HGSC through recruitment of TIL (tumor-infiltrating lymphocytes)	(27)
	n.a.	Effector T cells	- Intratumoral CXCR3 expression was upregulated in patients with advanced gastric and was associated with increased CD4^+^, CD8^+^ TILs infiltration and improved OS	(28)
	CXCL9 (MIG) CXCL10 (IP-10)	Effector T cells	- CXCL9, CXCL10 are important chemokines within the melanoma tumor microenvironment and are able to recruit CD8 effector T cells in a murine xenograft model	(29)
		Effector T cells	- CXCR3^−/−^ melanoma mice show accelerated tumor growth and impaired T cell infiltration of tumor tissue	(30)
		Effector T cells	- CXCR3 is essential for effector T cell trafficking through tumor vessels, even in absence of its ligands	(31)
	CXCL9 (MIG) CXCL10 (IP-10)	Effector T cells NK cells	- Human colorectal cancer samples show high CXCL9 and CXCL10 expression that correlates with T cell, but not NK cell numbers	(32)
	CXCL10 (IP-10)	NK	- CD27high CXCR3^+^ NK cells infiltrate tumors in murine lymphoma and melanoma models in an CXCL10-dependent fashion and lead to improved survival NK cells from CXCR3^−/−^ mice show impaired tumor infiltration	(33)
CXCR4	CXCL12 (SDF-1α/ß)	MDSC	- PGE2 increases CXCL12 levels in ascites of ovarian cancer patients - CXCR4^+^ MDSC are recruited toward CXCL12	(34)
		Treg	- CXCL12 levels are elevated in NSCLC, which results in increased recruitment of CD4^+^CD69^+^CXCR4^+^ T cells	(35)
		NK	- Genetically modified NK cells that overexpress CXCR4 lead to improved tumor eradication in a murine glioblastoma model	(36)

CXCR1 and CXCR2 are highly expressed by cytotoxic CD56^dim^ NK cells (37, 38). We recently showed that CXCR2 expression is downregulated on tumor-infiltrating NK cells in RCC and genetic modification to re-express CXCR2 enhanced recruitment of NK cells to the tumor site (39). Similarly, Ali et al. showed that CXCL8 was released within the TME of melanoma-infiltrated lymph nodes and could efficiently recruit highly cytotoxic NK cells (24). The percentage of this NK cell population among all NK cells within the affected lymph node was associated with improved prognosis among patients with stage III melanoma. Likewise, genetically modified CXCR2+ T cells displayed increased *in vivo* migration in murine melanoma models (40, 41). A clinical phase I/II trial in patients with metastatic melanoma infused with genetically modified CXCR2+ T cells has been initiated (Table 2).

**Table 2:** Clinical trials with modulators of chemokine functions within the tumor microenvironment.

**Name**	**Mode of action**	**Clinical trial**	**Current status**
**CHEMOKINE RECEPTOR ANTAGONISM**			
Reparixin	CXCR 1/2 inhibition	Phase IB (NCT02001974)	- Completed: 30% response rate in patients with metastatic breast cancer, well tolerated (42) - Combined with chemotherapy (paclitaxel)
AZD5069		Phase I/ II (NCT03177187)	- Recruiting patients with metastatic castrate-resistant prostate cancer - Combined with antiandrogen medication (enzalutamide)
SX-682		Phase I (NCT03161431)	- Recruiting patients with metastatic melanoma - Combined with immune checkpoint inhibitor (pembrolizumab)
AMD3100 (Plerixafor)	CXCL12/CXCR4 inhibition	Phase I (NCT03277209)	- Recruiting patients with pancreatic, ovarian and colorectal adenocarcinomas - Assess safety and impact on TME
		NCT02695966	- *Ex-Vivo* assessment of T lymphocyte function and localization in pancreatic cancer
Ulocuplumab (BMS-936564)		Phase I/II (NCT02472977)	- In combination with nivolumab - Terminated due to lack of efficacy
LY2510924		Phase II (NCT01439568)	- In combination with carboplatin and etoposide - No clinical benefit in patients with extensive-disease small cell lung carcinoma (43)
		Phase II (NCT01391130)	- In combination with sunitinib - Terminated due to insufficient efficacy in patients with metastatic clear cell renal cell carcinoma
		Phase 1 (NCT02737072)	- In combination with durvulumab for patients with advanced solid tumors - Terminated
USL 311		Phase I / II NCT02765165	- Recruiting patients with glioblastoma multiforme
Olaptesed (NOX-A12)		Phase I/II (NCT03168139)	- Olaptesed in combination with pembrolizumab - Recruiting patients with colorectal and pancreatic cancer
**GENETICALLY MODIFIED EFFECTOR IMMUNE CELLS**			
	CXCR2 + NGFR + T cells	Phase I/ II (NCT01740557)	- Recruiting patients with metastatic melanoma

Findings from pre-clinical studies have already been translated into clinical phase studies (summarized in Table 2). The combination of paclitaxel with reparixin—a CXCR1 and CXCR2 inhibitor—was well tolerated in patients with metastatic breast cancer and resulted in 30% response rate (42). Based on these findings, a phase II study was initiated (NCT02370238). Combination therapies with CXCR1/2 inhibitors are also in clinical phase trials for prostate cancer and metastatic melanoma.

## The role of CXCR3 and its ligands in solid tumors

CXCR3 is expressed on different subtypes of T and NK cells (37, 44) and binds to CXCL9, CXCL10, and CXCL11. During homeostasis, CXCL9, CXCL10 and CXCL11 are expressed at low levels by monocytes, endothelial cells and fibroblasts, but are upregulated upon cytokine stimulation, especially by IFNγ and TNFα (45, 46). CXCR3 and its ligands are expressed by various solid tumors, although their prognostic role greatly differs among the entities. This underlines a role in tumor suppression as well as tumor growth promotion and metastasis. While high CXCR3 expression in glioblastoma, colorectal, and breast cancer is associated with poor prognosis, it correlated with better outcomes in patients with gastric cancer (28, 47, 48). In contrast, high CXCL9, CXCL10, and CXCL11 expression in the TME of patients with colorectal, oesophageal, non-small cell lung (NSCL) and ovarian cancer is an indicator of improved overall survival (27, 49–51), while it is a poor prognostic marker in patients with localized clear-cell RCC (52).

CXCR3 is a key receptor in recruitment of activated T cells as it is absent in naïve T cells, but highly expressed on activated effector and memory T cells (44). CXCR3 expression on Tregs, however, can hamper effector immune cell functions due to competitive recruitment. In HCC, Treg infiltration in the liver after liver transplantation was associated with higher rates of recurrence (25). Patients with higher numbers of circulating Tregs and increased levels of CXCL10 within the graft were more susceptible. Similarly, high expression of CXCL11 in a colorectal cancer model was shown to recruit CXCR3^+^ Tregs (26). In contrast, in ovarian cancer, high CXCL9 and CXCL10 expression doubled the overall survival time due to improved recruitment of tumor-infiltrating lymphocytes (27). Enhanced effector T cell recruitment via the CXCR3 axis has also been confirmed in the case of gastric cancer and melanoma (28, 29). Tumor growth was accelerated in CXCR3^−/−^ melanoma-bearing mice and T cell infiltration was severly impaired (30). Anti-programmed death receptor (Anti-PD1) therapy was not beneficial in CXCR3^−/−^ tumor-bearing mice due to failure of efficient T cell recruitment (30). Importantly, CXCR3 has been shown to be indispensable for CD8^+^ effector T cell trafficking across tumor vasculature due to its role in intravascular adhesion, even in the absence of its ligands. CCR2 and CCR5, in contrast, promoted tumor site infiltration only in a chemokine ligand dependent manner (31). CXCR3 expression plays an important role in recruiting NK cells to the tumor site: We showed that CXCR3 expression on human NK cells increased during *ex vivo* culture (53). In xenograft mice models, these expanded NK cells could be efficiently recruited toward CXCL10^+^ melanomas (53). However, the sole presentation of CXCR3 ligands within the TME does not always predict efficient effector cell recruitment. In a mouse model of uveal melanoma that leads to spontaneous metastasis into the skin and viscera, application of the chemotherapeutic drug temozolomide increased CXCL9 and CXCL10 levels within the metastatic sites (54). Nonetheless, increased T cell infiltration was only observed in the visceral sites and not in the cutaneous tumors due to altered matrix architecture and mode of CXCL9/10 presentation (54). Interestingly, high expression levels of CXCL9 and CXCL10 in colorectal cancer samples correlated with T cell infiltration, but not with NK cell infiltration that was scarce in the analyzed samples (32). The expression level of CXCR3 was not measured on NK cells versus T cells. In contrast, CXCR3^+^ NK cells infiltrated tumor tissue in murine lymphoma and melanoma models in a CXCL10-dependent manner (33). CXCL10 was augmented via application of IFNγ (33). Several factors can modify CXCR3 expression on T cells and NK cells. For instance, elevated CXCR3 ligands in patients with cutanenous T cell lymphoma lead to CXCR3 downregulation on cytotoxic T cells (55). Soluble HLA-G was also shown to downregulate CXCR3 expression on cytotoxic T cells and inhibit migration along CXCL9 and CXCL10 gradients (56). In another study, STAT3 signaling in CD8^+^ T cells was shown to downregulate IFNγ production, leading to decreased CXCL10 expression by tumor-associated macrophages. Additionally, STAT3 diminished CXCR3 expression on CD8^+^ T cells (57). Collectively, these data underline not only the importance of the CXCR3 axis in recruitment of effector immune cells, but also reveal complex relationships of receptor-ligand interactions in a TME-specific context.

To enhance effector cell recruitment, efforts are made to increase CXCL9 and CXCL10 expression within the TME. Several enzymes can modulate CXCR3 ligands such as dipeptidyl peptidase-4 (DPP-4/CD26) (58, 59), furin (60) as well as certain peptidylarginine deaminases and matrix metalloproteinases (61). For instance, DPP-4 was shown to cleave CXCL9, 10 and 11, which in turn reduced their chemotactic activity on lymphocytes, while not affecting their antiangiogenic activities (59). In breast cancer cell lines, Prostaglandin E_2_ (PGE_2_) impaired IFN-γ mediated CXCL9 and CXCL10 release (62). Inhibition of the cyclooxygenase (COX) isoenzymes with indomethacin and acetylsalicylic acid suppressed the downregulatory functions of PGE_2_ and increased CXCL9 and CXCL10 levels *in vitro* (62). Evidence for the role of CXCL9 in attracting NK and cytotoxic T cells was shown in a murine model of breast cancer (63). Gene transfer of CXCL10 by pCLNCX retroviral vectors in melanoma xenograft models decreased angiogenesis and tumor growth (64). Similarly, murine-leukemia virus (MLV)-derived replication-competent retroviruses were used to stably express CXCL10 in fibrosarcoma, melanoma and Lewis lung cancer models and were shown to inhibit tumor growth *in vivo* (65). However, the effect of CXCL10 on T or NK cell recruitment and functionality was not investigated in these early studies. Only recently, an oncolytic poxvirus was armed with CXCL11 in order to attract CXCR3^+^ cytotoxic T cells and NK cells to the site of the malignancy in a murine mesothelioma model (66). Besides improving effector cell homing, the virus enhanced the systemic antitumor activity by inducing the proliferation of IFNγ-producing CD8^+^ T cells.

Targeting the CXCR3 axis to improve efficient effector cell recruitment is hampered by the opposing role on tumor cells: CXCR3 expression can be found on tumor cells, especially at later stages of tumorigenesis and in patients with advanced disease, where it is positively correlated with the formation of metastasis (67–69). Thus, blocking CXCR3 on tumor cells might also impair the ability of CXCR3+ NK and T cells to efficiently kill tumor cells. Interestingly, ACKR3 (formerly CXCR7) is an atypical receptor of CXCL11 and CXCL12, that is not expressed on peripheral blood leukocytes but upregulated by various tumor types, including breast, esophageal and lung squamous cell cancer (70, 71). Targeting of ACKR3 with a monoclonal antibody in mice models of glioblastoma leads to increased tumor cell death via NK-cell mediated antibody-dependent cytotoxicity (ADCC) (72). Combination with temozolomide prolonged survival in tumor-bearing mice and resulted in enhanced infiltration of anti-tumorigenic M1 macrophages (72). CXCR3 and ACKR3 inhibitors are in preclinical testing for different solid tumors (72–74). Currently there are no registered clinical phase trials employing either CXCR3 or ACKR3 inhibitors in solid malignancies.

## CXCR4 and its ligand CXCL12

CXCR4 and its ligand CXCL12 are ubiquitously expressed under physiological conditions and are important for hematopoiesis, cardiogenesis, and neurogenesis. The CXCR4-CXCL12 axis is involved in HSC maintenance and homing within the bone marrow as well as during the development of B, T, and NK cells (75, 76). In the context of cancer, CXCR4 expression is found on tumor cells, where it promotes tumor cell growth, migration, and invasiveness (77, 78). Moreover, CXCL12 produced within the tumor can attract CXCR4^+^ Treg, MDSC and plasmacytoid dendritic cells (pDC), potentiating the tumor-promoting effect (34, 79–81). High CXCR4 expression in biopsies of solid tumors is generally correlated with worse prognosis. In particular, CXCR4 expression in breast cancer was significantly associated with lymph node and distant metastasis and worse overall survival (82). Similar conclusions could be drawn for prostate cancer, melanoma and lung cancer (83–85).

The expression levels of CXCR4 on NK and T cells varies according to their maturation stage and subset, whereas their recruitment to the different organs is often dependent on the co-expression of other chemokines (86, 87). High CXCR4 expression on NK cells is associated with accumulation within the bone marrow compartment, whereas CXCR4 desensitization is important to enable NK cells to leave the bone marrow (88, 89). Several factors can modulate the chemokine receptor repertoire on immune cells: For instance, conditioning human NK cells with TGFβ1, derived from neuroblastoma cells, significantly upregulated CXCR4 and CXCR3 expression and downregulated CX3CR1 on NK cells (90). This generated an NK cell phenotype that is retained in the bone marrow, rather than recruited to peripheral organs and tumor tissue (91). Another study suggested that PGE2 regulates CXCL12 levels in malignant ascites from ovarian cancer patients and CXCR4 expression on MDSC (34). Blockade of PGE2 abrogated migration of MDSC toward the malignant ascites. In line with this, non-small cell lung cancer (NSCLC) express high CXCL12 levels and especially recruits CD4^+^CD69^+^CXCR4^+^ T cells with an increased ratio of regulatory T cells (35). Although the percentage of CD8^+^ T cells was not altered, NK cell numbers within the tumor tissue decreased. In accordance, regulatory T cells are maintained within the bone marrow and can migrate along the CXCR4-CXCL12 axis (92). Regarding modulation of CXCR4 expression using pharmacological agents, tyrosine kinase inhibitors (TKIs) imatinib and nilotinib have been shown to selectively increase the cell surface of CXCR4 on NK cells and monocytes, *in vitro* experiments using NK cells derived from neuroblastoma patients (93).

Multiple approaches to target this axis have been explored, some of which have entered clinical trials with varying outcomes (summarized in Table 2). On a preclinical level, TN14003 and AMD3100 (Plerixafor), two anti-CXCR4 inhibitors, have been tested in patient-derived xenografts (PDX) of breast cancer showing antitumor activity in the HER2 subtype (94). Interestingly, in triple-negative PDX, both inhibitors appeared neither to control tumor growth nor to impede metastatic spread, which highlights the complexity of breast cancer subtypes and their respective TMEs. AMD3100 has also been tested in a murine model of human pancreatic cancer, alone or in combination with immunological checkpoint antagonists (95). In this study, AMD3100 was able to successfully block CXCR4 signaling and promote T-cell mobilization *in vivo*. More importantly, AMD3100 showed improved anticancer activity when combined with an anti-PD-L1 monoclonal antibody (96). CXCR4 is also highly expressed in colorectal cancer, building a therapeutic rationale for CXCR4 targeting (97). Blocking colon carcinoma cells with a CXCL12-KDEL retention protein *in vitro*, resulted in the inhibition of CXCR4-mediated signaling and a subsequent dramatic decrease in metastatic cancer outgrowth (98). AMD3100 has also been tested in the particular model, exhibiting similar promising preclinical results (99). Other means of modulating the CXCR4-CXCL12 axis include oncolytic viruses and gene-engineered NK cells. In particular, introducing an oncolytic virus equipped with a CXCR4 antagonist restored the pathologic signaling in a murine model of ovarian cancer, reduced metastatic spread and diminished regulatory T cell recruitment (100). On the other hand, NK cells engineered to co-express a chimeric antigen receptor (CAR) and the chemokine receptor CXCR4 enhanced NK cell infiltration and tumor cell killing in a glioblastoma tumor model (36). Last but not least, Spiegelmer aptamers, such as the CXCL12-targeting NOX-A12, hold great potential in modulating the TME of solid tumors. Although clinical trials are still ongoing (Table 2), NOX-A12 (Olaptesed pegol) is thought to increase immune cell infiltration, sensitize tumors to checkpoint inhibitors and obstruct tumor repair mechanisms in metastatic pancreatic and colorectal cancers (Noxxon Pharma). Examples of additional types of solid tumors that may benefit from inhibition of the CXCR4-CXCL12 axis are oesophageal (101)and gastric cancer (102).

## Concluding remarks

Although our current understanding of solid tumor microenvironment and its chemokine networks is more detailed, a lot remains unexplored. The future of chemokine modulation for therapeutic purposes is very much dependent on efforts to elucidate the complex pro-tumor and antitumor roles of chemokines in the TME. The current preclinical approaches have demonstrated some promising results and defined rational immunotherapeutic combinations. The results from the eagerly awaited clinical trials, in combination with investigations on new chemokine targets and advances in drug discovery, immunotherapy and cell therapy, are expected to shape the landscape of chemokine-based therapy further in the years to come.

## Author contributions

All authors listed have made a substantial, direct and intellectual contribution to the work, and approved it for publication.

### Conflict of interest statement

The authors declare that the research was conducted in the absence of any commercial or financial relationships that could be construed as a potential conflict of interest.
